# ATIPS: Automatic Travel Itinerary Planning System for Domestic Areas

**DOI:** 10.1155/2016/1281379

**Published:** 2015-12-29

**Authors:** Hsien-Tsung Chang, Yi-Ming Chang, Meng-Tze Tsai

**Affiliations:** Department of Computer Science and Information Engineering, Chang Gung University, 259 Wen-Hwa 1st Road, Kwei-Shan, Taoyuan 333, Taiwan

## Abstract

Leisure travel has become a topic of great interest to Taiwanese residents in recent years. Most residents expect to be able to relax on a vacation during the holidays; however, the complicated procedure of travel itinerary planning is often discouraging and leads them to abandon the idea of traveling. In this paper, we design an automatic travel itinerary planning system for the domestic area (ATIPS) using an algorithm to automatically plan a domestic travel itinerary based on user intentions that allows users to minimize the process of trip planning. Simply by entering the travel time, the departure point, and the destination location, the system can automatically generate a travel itinerary. According to the results of the experiments, 70% of users were satisfied with the result of our system, and 82% of users were satisfied with the automatic user preference learning mechanism of ATIPS. Our algorithm also provides a framework for substituting modules or weights and offers a new method for travel planning.

## 1. Introduction

Given current levels of living standards and incomes, Taiwanese citizens now pay more attention to the quality of their leisure life [1]. Many families take advantage of holidays and summer and winter vacations to arrange simple travel itineraries or multiday travel plans. According to the statistics of the Taiwan Tourism Bureau [2], among the many possible vacation styles for Taiwanese residents, self-planned trips account for the highest proportion. The statistics show that many people prefer to create custom-made trips that meet all of their expectations. However, to plan an itinerary, travelers must invest time and effort in advance. Therefore, a system that arranges personal travel itineraries, provides options for dining, entertainment, and accommodation, and uses a simple and quick procedure will reduce the time that it takes vacationers to schedule their travel.

Based on our observations, travel styles can be classified into two types: the first involves moving from a departure point to a destination that provides entertainment and the second involves starting from a departure point and considering the journey to be part of the entertainment. In either travel type, self-planned travel is still the favorite option for the Taiwanese people. However, planning your own travel first requires finding extensive information, perhaps by using keywords on the internet to search for tourist destinations recommended by netizens. Tourist spots that are popular on search engines will have a large amount of information available.

After identifying interesting travel destinations, the relevant tourist destinations, dining places, and hotels must be consecutively reserved for a smooth transition during the entire trip. However, these types of procedures may be complicated; therefore, this study involving ATIPS provides an algorithm to automatically plan a domestic travel itinerary based on user intentions that allows users to minimize the trip-planning process. Simply by entering the travel time, the departure point, and the location of the destination, the system generates a travel itinerary. Based on the above information entered by users, feedback from user's previous travels using the system, and user's traveling habits, a travel itinerary matching user's preferences is automatically generated. This research will focus mainly on the travel mode that involves starting from a departure place and finding entertainment while travelling to the destination.

Based on the researches [3], they map the travel needs for Ambient Assisted Living (AAL) into three parts which are planning before the travel, tracking during the trip, and assessment after the trip. The recommendation and planning functions play an important role before the trip in the AAL. ATIPS provides the recommendation and planning functions to help people reduce the efforts before the trip.

## 2. Related Works

Researchers have previously proposed methods for automatic itinerary planning. In “A Travel Scheduling System based on Spot Evaluation” by Masanobu and Kohtaro [4], the concept of classifying the characteristics of a tourist spot according to different properties, for example, culture and history, was proposed. After classifying the tourist spots into different categories according to their properties, the system asks the user questions related to the region to assess their impression of the region or their interest in history, culture, and nature. Based on the answers, a score is given to each tourist spot, including restaurants and hotels, and several itinerary options that rank the tourist spots in accordance with their scores are provided. 

Shiraishi et al. [5] and Maruyama et al. [6] converted the search process to produce a travel itinerary that was a multiobjective optimization problem. In their study, different travel schedules are first randomly generated, and the optimal travel schedule for users is then selected from these schedules. The tourist spots in the perfect travel itinerary must simultaneously match the users' intentions and time restrictions; the distance between the tourist spots of interest cannot be too far, and other factors, such as cost, should also be considered. The suitability of each itinerary is obtained by a series of calculations that incorporate these factors, and this is then used as the selection score.

Maruyama et al. [6] proposed another method for scheduling travel. First, the user is asked to enter a series of tourist spots of interest, including the name of the tourist spot, the degree of preference, and the estimated time of arrival. Then, the input data are transferred to the server. The server searches for and selects the shortest path between any two tourist spots using the A^*∗*^ algorithm. In the second step, the reproduction, crossover, and mutation of the genetic algorithm (GA) [7] are performed, that is, the 1st generation evolution. After a certain number of such evolutions, the travel itinerary with the highest final score is selected as the itinerary for the trip.

Sebastia et al. [8] and Garica et al. [9] proposed the Recommender System (RS) to recommend tourist locations to users. A first-time user of this system must input some relevant information, including the type of desired tourist spot, with the associated degree of each preference. In each subsequent use of this system, the user must input the types of tourist spots desired for the current trip with the degree of preference. The system will select a series of tourist spots according to the information given during user registration, the information input for the current trip, and the preferences of other users. Xie et al. [10] proposed a planning method for travel to tourist spots that also helps users to quickly identify destinations using the RS. Unlike the method proposed by Garica et al., Min's system selects the top *k* tourist spots with the highest scores among the assessed spots in the many-component RS and external database to form a package, which is the travel itinerary. A place of interest (POI) refers to a tourist destination that current users may find interesting and that can be used within travel plans. Therefore, there are many POIs in each package, and every POI in the package should satisfy the upper limit of cost and any time restrictions. The user will select a favorite from these top *k* travel schedules as the itinerary for the current tour.

In these earlier studies [11], the methods for itinerary planning either retrieve all tourist spots in a city to find the shortest path or list the recommended tourist spots to users so that they can make their own itineraries. The former methods can provide users with the shortest path of travel, but the tourist spots in the trip may not be of the most interest to the users. The latter systems identify tourist spots that are closely related to the user's interests and recommend them. However, after obtaining the best tourist spots, users still must find the shortest or smoothest path without detours. An overview of the two travel itinerary planning methods that are currently available shows that neither can simply and quickly provide an itinerary for users that meets their needs. Therefore, this study proposes a method that achieves this objective: simply and quickly selecting tourist spots for different users and automatically generating a travel itinerary consisting of those that meet user's indicated preferences.

## 3. Design and Implementation of ATIPS

**Figure 1:**
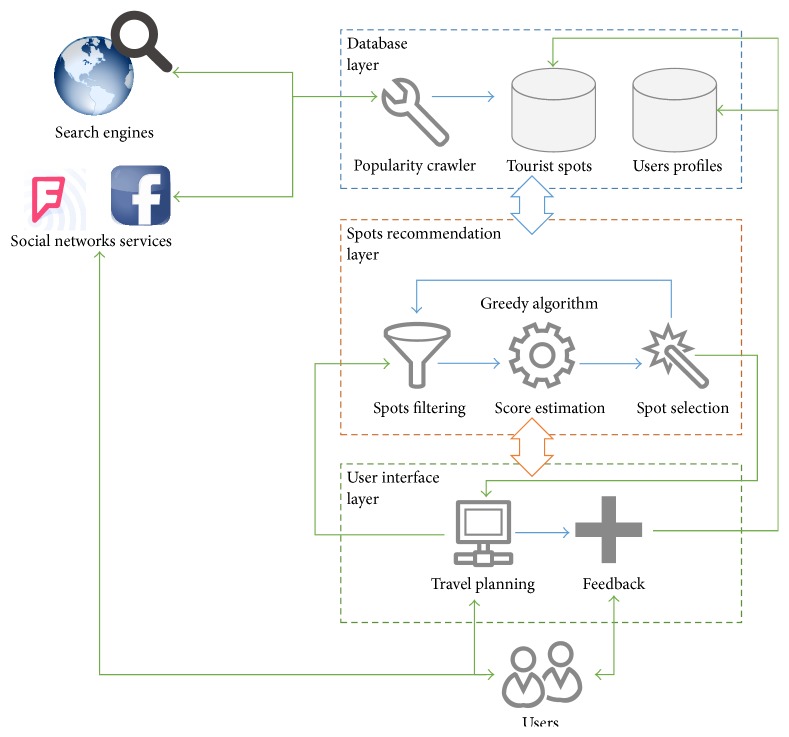
System architecture of our proposed ATIPS.

This section describes the source of information for tourist locations and how it is stored. It will also introduce the actual operating procedure of the system, the step-by-step user guide, the quality evaluation of the information, and the quality evaluation for each tourist spot.  Figure 1 shows a diagram of the system architecture, which has three layers. The first is the database layer, which stores the data for tourist spots and users; the second layer is the spot recommendation system, which receives the requests and processes the data from the database layer; and the last layer is the user interface layer, which interacts with users and collects feedback for the database layer.

A first-time user of the system must register an account, which only requires a username and password to log into the system. When the user wants to use the system to plan a trip, the addresses for the departure point of the trip and the destination need to be entered first; using the departure address, the system will search for nearby tourist spots and calculate the score of each spot. This system applies a greedy algorithm that takes the most favorable choice from all currently available options for every step, and the final result is expected to be the most favorable [12]. The tourist spot with the highest score is selected to be the next tourist spot in the itinerary, and the itinerary gradually builds toward the destination input by the user.

### 3.1. Tourist Spots

There are many sources of information for tourist spots, especially considering the numerous services that are now available on the web. To increase its effectiveness, our system also uses commercial travel books as an information source. The information for the tourist spots included in travel books includes the history of the destination and describes, in detail, the characteristics of each location that are worth special attention. In addition, books also provide the address of each tourist spot, as well as the telephone number and the geolocation, that is, the latitude and longitude. If the store or the tourist spot cannot be found using the address, the latitude and longitude information allows travelers with a global positioning system (GPS) to accurately locate it, and the store can also be contacted over the telephone.

### 3.2. Data Storage

**Figure 2:**
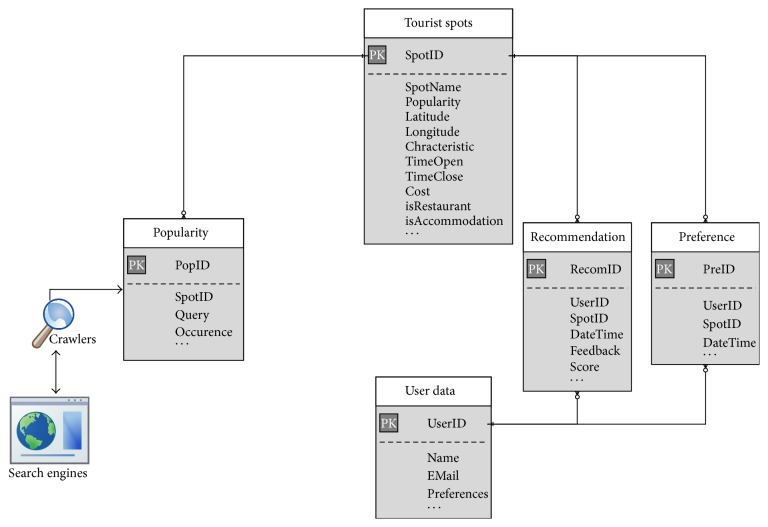
Data schema in ATIPS database system.

Such a large amount of rich information is difficult for the system to assess in its raw form. Therefore, data processing is necessary so that the information can be simply and accurately used to generate itineraries that meet the needs of the users and that include all their favorite tourist locations. In addition to recording the name, location, and time of operation as provided in travel books, other characteristics of the tourist spots are also recorded, as shown in Table 1. We use a MySQL database management server to store the information on tourist destinations, user data, preferences, recommendations, and popularity, as shown in Figure 2.

ATIPS stores not only information related to the tourist spots but also information regarding the travel habits of different users. When users register on the system, they assign initial preference scores to each tourism category. ATIPS will subtract a value *α* from the corresponding preference score of a specific location every time a user removes the location from a travel itinerary. Considering one removed tourist spot, the spot might be removed for many different reasons. However, as an increasing number of travel plans are performed by ATIPS, the feedback from removed tourist spots could be incorporated into the user's preference. For each location not removed by the user, the system will add a value *β* to the corresponding preference score. For example, Taoyuan Martyrs' Shrine is included in the travel itinerary generated by the system. If the user removes this location from the trip, the system will recognize that this user is not interested in the category of history and culture as represented by Taoyuan Martyrs' Shrine; therefore, the preference score for history and culture of this user will be different from the initial value. This system can thus learn the travel habits of users. When the user tries to plan a future trip using our system, the resulting itinerary will increasingly reflect the user's preferences.

### 3.3. Planning the Travel Itinerary

Based on our observations, when tourists are travelling, they usually travel to a location and then find popular and interesting places nearby to visit while there or during the trip. This habit is similar to the strategy of first selecting the best attraction in an area as the destination and then searching for other tourist spots to visit one by one. The ultimate purpose of a greedy algorithm is to solve a problem by dividing it into many smaller steps. After the current optimal solution is calculated at each step, the algorithm can then seek the resulting optimal or nearly optimal solution after the entire problem is linked together. The strategy that a greedy algorithm uses to solve a problem is the same as the travel habits of most people; that is, people usually visit the interesting tourist spots near them. Based on this concept, the system calculates the scores of nearby attractions and selects the one with the highest score as the next one to visit. Then, the system continues searching for subsequent attractions from the current position until the destination is reached.

**Figure 3:**
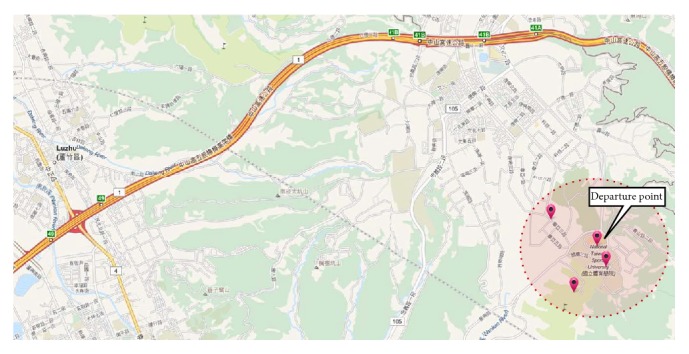
Capturing the tourist spots around a departure point within a predefined distance (map copyrighted by ©OpenStreetMap contributors, CC BY-SA).

As shown in Figure 3, ATIPS takes the departure point entered by the user during the itinerary planning step as the “start.” Taking the “start” as the center, all tourist spots within a certain radius are captured. The system calculates the score for these destinations and evaluates them based on the calculated scores. The destination with the highest score is included in the trip. After the position of the first destination is obtained, the next is calculated with the same method using the first tourist spot as the center. The subsequent tourist stops are calculated one-by-one using this method to gradually move toward the end point, a process that results in the formation of a travel itinerary. However, there are some issues that must be resolved when using this concept; for example, how do you ensure that this method will reach the destination? And how do you compute the score for each tourist spot? We introduce five factors to resolve this problem: user preferences, popularity, cost, distance, and time. These will be described in the following and used to allow ATIPS to solve the problems. The initial value of each vector can be set manually in the beginning stage of the ATIPS system. The value can be dynamically adjusted according to user feedback to generate a more accurate score for each tourist destination.

#### 3.3.1. Characteristics of the Tourist Spots and User Preferences

We will use five factors to calculate a score for each tourist spots captured. Among these five factors, the most important is user preference. ATIPS will automatically generate a travel itinerary according to the user's preferences, so the system must search for attractions that match user interests when selecting stops in the itinerary. The first consideration is whether the characteristics of the attraction match the user preferences. Defining *Sp*_*i*_ as the score of tourist spot *i*, *S*_*i*_ as the vector associated with the tourist spot *i*, and *P*_*u*_ as the vector of the user preferences, the score is calculated using (1), where *i* refers to the *i*th tourist spot and *u* refers to a user, as follows:

(1) $$\begin{array}{c} {Sp}_{i}=S_{i}\cdot P_{u}. \end{array}$$

*S*_*i*_ represents the vector value of the tourist spot as shown in (2), where *k*, *e*, *n*, and *c* represent the properties of intellectual interest, recreation, natural ecology, and history and culture of the attraction, respectively, and *P*_*u*_ is the vector of user preferences, as shown in (3). For the tourist spot vector *S*_*i*_, the value of each variable in the vector ranges from 0 to 1, representing the relevance of the tourist spot with respect to the associated property; that is, 0 indicates that this tourist spot does not have the property, and 1 indicates that the attraction matches the user preference in this property. For example, the tourist spot vector *S*_*i*_, which is (0.7, 0.3, 0, 0), indicates that this tourist spot has the characteristics of intellectual interest and recreation. For the vector of user preferences, *P*_*u*_, each property has a score, and each score represents the degree of preference user *u* has with respect to this property; the maximal value is 10. The score of a tourist spot is the combination of scores for the various properties. The score calculation for the properties of a tourist spot is the combination of whether this destination has a particular tourist property and the preference of the user for this property, which can be obtained as the inner product of the tourist spot vector, *S*_*i*_, and the vector of the user preferences, *P*_*u*_. The value of *S*_*i*_ was initially set manually, and it can be dynamically adjusted according to user feedback to apply the impact of social networks. Consider

(2) Si=ki,ei,ni,ci,

(3) Pu=ki,ei,ni,ci.

#### 3.3.2. Popularity

The second factor is popularity. Tourist destinations with a higher level of popularity will attract more tourists. The popularity of a tourist spot is obtained from the number of times this destination has been included in users' travel itineraries or the recommendation score from users. This information becomes more accurate as the system operates longer and gathers more data. Our newly established system does not yet contain complete information on the popularity of tourist spots. Therefore, the results from a search engine serve as the basis for the initial assessment of popularity in this study. For example, performing a keyword search using the Google search engine will provide the number of results for that keyword, which represents the number of available documents containing the keyword. A compilation of the search results for each tourist spot will result in significantly different numbers, ranging from several thousands of documents to several millions or even more than tens of millions of documents. To present the values in a closer range to avoid too broad a dispersion, the data from the search results are adjusted, as shown in (4). *Sh*_*i*_ is defined as the popularity score of a tourist spot, SpotScore_*i*_ represents the search result for tourist spot *i*, and MaxScore represents the highest number of search results. Both the original value and the adjusted value, that is, the value normalized to a range from 0 to 10, of the search results for each tourist spot are saved in the database. The value of *Sh*_*i*_ could easily be replaced by a recommendation score from social networks or a count of visits to tourist spot *i* to generate more accurate estimations. For example, we can combine ATIPS to the “likes” function in Facebook to be associated with the tourist spots; this could be treated as the recommendation feedbacks from users. The numbers of “likes” and “check in” from Facebook and Foursquare to a spot *i* could be the sources to calculate the popularity value *Sh*_*i*_ for the spot evaluation. Consider

(4) $$\begin{array}{c} {Sh}_{i}=10*\left(\;\frac{{\text{SpotScore}}_{i}}{\text{MaxScore}}\;\right). \end{array}$$

#### 3.3.3. Cost

The third reference factor is cost. Some tourists are less concerned about the cost of travel. However, others are more careful about spending. Therefore, users are required to provide an evaluation of their cost concerns during the registration process. Consider

(5) Sci=Cu∗costi,

(6) costi=10∗1−spentimaxSpent.

As shown in (5), Sc_*i*_ represents the score of the cost for tourist spot *i*, and *C*_*u*_ represents the degree of concern of user *u* regarding costs incurred during travel, which is a value between 0 and 1. As shown in (6), spent_*i*_ is the spending at tourist spot *i*, maxSpent represents the highest spending amount, and cost_*i*_ is the normalized value between 0 and 10. For future travel planning using the system, the score of the cost factor will be calculated using (5).

#### 3.3.4. Distance

The fourth reference factor is the distance, *Sd*_*i*_. Distance can be divided into two parts. The first part is a calculation of whether the location is within the radius of the reference point according to its Euclidean distance from latitude and longitude. The goal of the greedy algorithm is to identify the best tourist spot at the current stage; therefore, we require the tourist spot selection to be within a certain radius of the current position. Tourist spots beyond that radius are not included for consideration. Without a radius restriction, too many scores will be calculated, most of which will be useless. To avoid the unnecessary calculation of scores, at the initial stage of selection, the system will calculate the distance from the current position to each tourist spot. If the distance exceeds the radius defined by the system, this tourist spot will not be selected. The second part of the distance calculation is performed for attractions within the radius according to the path distance as generated by the Google API. In addition to using the radius to avoid the unnecessary calculation of scores, the difference between the path distance from the current position to the destination and the path distance from a tourist spot to the destination is also considered as a reference. If the difference between the two distances is negative, it can be assumed that this location may not be on the route to the destination, and the score is adjusted accordingly. If the difference between the two path distances is too large, the score of the distance factor will be greatly reduced. This design will ensure that our planned trip moves toward the destination. However, our score calculation is a multifactor equation: if the score of other factors is high enough, the tourist spot could be selected into the itinerary even if distance value is negative.

#### 3.3.5. Time

The last factor to be considered is the time factor, *St*_*i*_. The time factor will determine the itinerary fate and the distance score. The goal is for the user to be entertained while traveling toward the destination. At the beginning of the journey, the system will select tourist spots relatively close to the departure point. As the tour progresses to the middle of the trip, the system will try to select tourist spots close to the midpoint between the start and destination. When the journey is close to its end, the score for tourist spots near the destination will be increased. The previously calculated difference between the distance from the current position to the destination and the distance from the tourist spot to the destination is multiplied by the ratio of the time used to the total traveling time. According to (7), the longer the traveling time, the higher the distance factor score. These decisions will adjust the entire distance scores within the total overall score over time. Some tourist spots are time sensitive, such as the operating hours of an amusement park or the time that a night market is open. If a trip's departure time is at 9:00 am and a night market usually opens at 4:00 pm or 5:00 pm, the score calculation at this stage should identify that the night market is not yet open and that it and other tourist spots with unmatched open times must be excluded. That is, the system will avoid making calculations for tourist spots with hours of operation that do not allow users to visit in order to reduce unnecessary calculations. The current time is calculated as the time spent at each location plus the driving time. The system will assign different times to the different attractions according to their characteristics. The driving time is calculated as the straight-line distance between the two locations divided by the driving speed, which is chosen as the average speed limit set by the National Freeway Bureau, Taiwan's Ministry of Transportation [13], that is, 100 km per hour.

The overall score for a tourist spot can be calculated using (7). Each factor is multiplied by a weight value, which represents the proportion of this factor in the score of the tourist spot. The sum of all weight values is 1. For each score for a tourist spot, the score of each factor must be calculated individually. Then, each factor is multiplied by its corresponding weight value, and the scores are summed. The final resulting total score is the score of the tourist spot for the current user. The entire process for travel scheduling is shown in Algorithm 1.

As shown in Algorithm 1, the system first determines whether dining or sleeping is appropriate for the current time. If it is time to eat or sleep, then the system locates a restaurant or accommodation; otherwise, it will search for tourist spots and recommend them to the user.  Algorithm 2 shows the procedure for finding attractions, in which dist(cp, *s*) is the distance between the current position, cp, and the attraction, *s*. Therefore, our system will first search all tourist spots within the radius; then, the scores for those within the radius range are calculated, and suitable destinations are recommended to the user based on the scores.

After the scores of the five factors are calculated, the scores for all tourist spots are determined, and the travel itinerary is arranged for the user according to these scores. As mentioned earlier, the user can remove a tourist spot from the system-generated itinerary if it is not satisfactory, and the system will proportionally adjust the scores for that user's preferences based on this feedback. The system will also be able to recognize the retained tourist spots, and the corresponding scores of the user preferences will be increased by *β*. Regardless of how the score is adjusted, the total scores of the user preferences will always be controlled within a fixed number. The calculation of the parameters *a*, *b*, *c*, and *d* in the score will be defined in Section 4. Consider

(7) $$\begin{array}{c} E_{i}=a*{Sp}_{i}+b*{Sh}_{i}+c*{\text{Sc}}_{i}+d*{Sd}_{i}*{St}_{i}. \end{array}$$

Our algorithm in (7) is a framework that other systems can use to substitute their modules or weights to become a new method for travel planning.

## 4. Experiments and Discussions

Our experimental data sources include three travel books: Outdoor Life: Weekend Car Travel in Pei Kee Yi [14], Weekend Car Travel in Tao Tsu Miao [15], and Weekend Car Travel in Central Taiwan [16]. The number of tourist spots in the database was expanded using the tourist spots introduced in these travel books. Our experiments and the tourist spots include Keelung City, Taipei City, New Taipei City, Taoyuan City, Hsinchu County, Miaoli, and Taichung. ATIPS could be operated in other geographic settings by increasing the information regarding tourist spots for domestic area travel itinerary planning. In addition to the ATIPS algorithm, the data on tourist spots also serves as an important role in the system.

### 4.1. Travel Scheduling

Many options for travel software or mobile applications have been released recently, as shown in Table 2. They provide information regarding tourist spots and itinerary planning tools for users. However, users need to select the spots manually, and these systems do not offer any learning of preferences. Our proposed system, ATIPS, automatically plans a travel itinerary and learns preferences automatically during the interactive adjustment of this itinerary.

**Figure 4:**
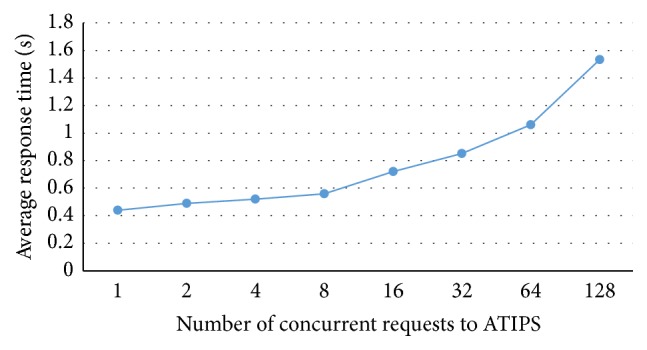
Average response time with different number of concurrent requests to ATIPS.

The ATIPS system is designed to be a web service, so it can be used on different platforms. It can also be easily transferred into mobile apps directly or using the ATIPS API. Our system performs SQL queries to the mysql server to generate results for users. We conduct an experiment that utilized a web crawler connecting to ATIPS simultaneously for travel itinerary planning with different number of requests.  Figure 4 demonstrates the average response time in second according to the different number of requests. The average response time is less than two seconds even simultaneously sending 128 requests to our system. The response time is reasonable for a web service.

To increase the usability of ATIPS, we designed the system to be simple to use. The procedure for travel scheduling uses a common user interface of the web service, and it should be as simple as possible so that users will continue to use the service. Therefore, to register on this system, the user only needs to input a username, a password, and an estimation of his/her level of concern regarding the cost of travel.

**Figure 5:**
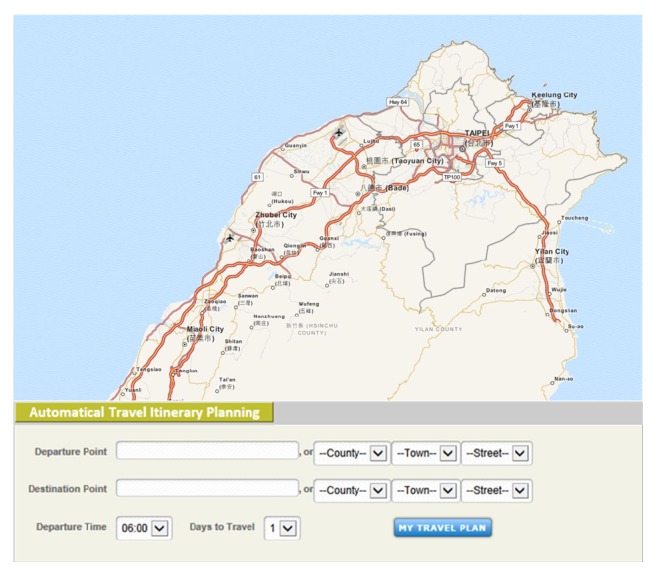
A snapshot of ATIPS before travel scheduling (map copyrighted by ©OpenStreetMap contributors, CC BY-SA).

**Figure 6:**
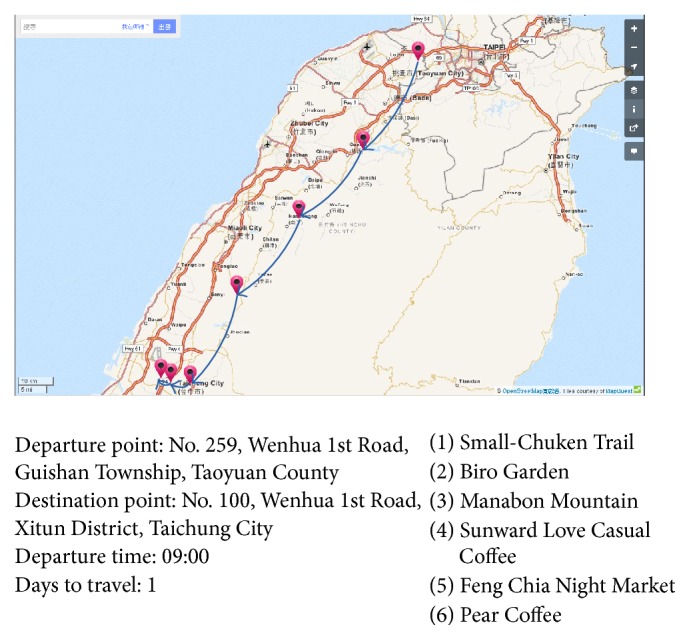
The results from the travel planning algorithm (map copyrighted by ©OpenStreetMap contributors, CC BY-SA).

Both the registration procedure and the procedure for travel scheduling are simple for users, as shown in Figure 5. After logging on the system, the user enters the locations of the departure point, which could be selected as the current GPS location, and the destination point; these two points allow the system to understand how to arrange the entire travel itinerary from beginning to end. The user also inputs the desired starting time, which allows the system to accurately select dining establishments for the user. For a multiday tour, the system can also accurately select accommodations by location based on user's starting time. While scheduling travel, the user also indicates the number of days for travel, and the system will establish a travel schedule according to the method described in Section 3. The final travel itinerary plan is presented to the user as shown in Figure 6, with the complete location information for all selected tourist spots, the tour roadmap, and the trip directions.

Because different users have different preferences, for newly registered users, the system does not know a priori their preferences or favorite style of travel. Therefore, the system will ask new users to input initial preferences for different types of tourist spots. Thus, after registration, newly registered users must provide scores for the four travel categories of intellectual interest, recreation, history, and culture and natural ecology to indicate their preferences. Subsequently, after each travel scheduling session, the system will be able to determine whether the user is satisfied by using the feedback information, which includes the deletion of tourist spots by the user. If the user finds a tourist spot uninteresting and removes it, the system will identify the category of that removed tourist spot and subtract *α* from the category for this user. This mechanism for automatically learning user preferences can eventually increase the usability of ATIPS.

Therefore, if a user uses the travel planning system frequently, the system will learn the travel preferences of that user as he or she deletes destinations in the travel plan. Furthermore, the system can design a customized route matching the preferences for each user.

### 4.2. Heuristic Evaluation for Usability

Heuristic Evaluation (HE) [23, 24], which was proposed by Nielsen, was applied in this paper. HE uses ten usability evaluation principles to identify potential interface usability issues. We invited three experts in user interface design, social network, and computer science to participate in the usability assessment of the ATIPS, including two experts with rich experience in information and social network system design and one expert with a strong background in interface design and usability. The experts were asked to assume the role of a general user for system evaluation. We recorded whether the experts considered the system to present usability issues for each criteria in the inspection process. We list the usability evaluation principles with comments from experts as follows:

- *User control and freedom*: there is no sufficient navigation buttons in ATIPS. Experts recommended including navigation buttons to increase the user control and freedom.
- *Error prevention*: no error prevention function is provided. When a user deletes a tourist spot in an error, no undo function is provided. The three experts recommended including error prevention and undo functions in ATIPS.
- *Help users recognize, diagnose, and recover from errors*: ATIPS does not provide the user manual, it is better to include help dialogues to help users get rid of errors.
- *Help and documentation*: ATIPS does not provide user manuals and guides. The three experts recommended including simple instructions to help new users quickly learn to operate the ATIPS.

### 4.3. Experimentation

The proposed method of automatic itinerary planning based on user preferences is different from the general method of travel planning. Generally, when people plan their travel itinerary, they reference the most popular and frequently discussed attractions. However, tourist spots that are more popular and that are discussed by more people are usually biased toward a single county or city. In addition, it is difficult for a self-planning traveler to simultaneously consider a variety of factors such as cost and time. If travel itinerary planning only considers the number of discussions about a tourist spot, its popularity, or whether it is on a search engine's ranking list, the resulting route determined by this planning method will not consider user's interests or other important factors. A travel plan also requires additional itinerary planning to provide directions or the visiting order of the attractions. These factors require a large amount of time and effort, which may discourage Taiwanese residents with little travel experience from taking a trip for a vacation.

The proposed approach integrates many factors affecting tourism that have been proposed in the literature, calculates the score of each tourist spot including the impact of cost and time, and selects the target tourist spots in the itinerary according to their scores. Therefore, the method for calculating tourist spot scores is relatively objective and is also better than travel planning methods based solely on popularity.

Five college students were recruited to participate in a test of the parameters of our score calculation equation. As defined, the sum of the four parameters, *a*, *b*, *c*, and *d*, in the score calculation equation must equal 1; that is, the parameters are under certain restrictions. Each of the five college students was asked to manually arrange a trip itinerary that they thought was perfect. Then, all possible combinations of *a*, *b*, *c*, and *d* were calculated and introduced into the equation to calculate the scores of all tourist spots in the arranged trip, and these calculated scores were summed. For each participant, the set of parameters with the highest overall score for all tourist spots was selected as that participant's parameter set. The parameter sets for the five participants were averaged to obtain the best combination of parameters *a*, *b*, *c*, and *d*, which are 0.05, 0.05, 0.1, and 0.8, respectively. The subsequent score calculations with the equation were based on this set of parameters.

**Figure 7:**
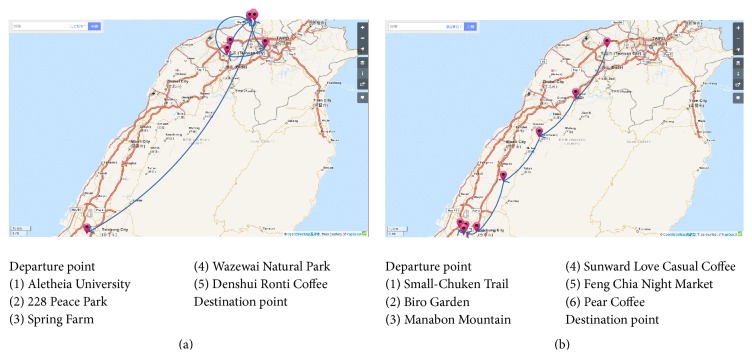
Comparison of the total scores obtained using the different methods (map copyrighted by ©OpenStreetMap contributors, CC BY-SA).

Figure 7 shows two methods for itinerary planning. The method on Figure 7(a) is a general method often used in itinerary planning that only considers the popularity of tourist spots in the itinerary. The method used in Figure 7(b) is that used in the proposed system and is based on user preferences and considers other factors. Several travel itinerary planning sessions were completed using these two methods, and the scores for the tourist spots in each itinerary were calculated and summed. The total overall scores for all itineraries with each method were averaged and compared.

In the traditional system, popularity is the most important factor; the following experiment tries to compare the difference between using only the popularity factor and using all factors. The results in Table 3 show that when the travel plan is only based on the popularity of the tourist spots without considering other factors, the average scores of the tourist spots are lower than those generated using our proposed method. Thus, our method not only effectively plans a travel itinerary but also considers various factors for each tourist spot, resulting in an overall optimal travel itinerary that includes all selected tourist spots.

The performance evaluation of travel itinerary planning is a subjective judgement. It is hard to use classic information retrieval metrics, such as recall and precision, to evaluate ATIPS. In the following experiments, we asked participants to use our systems with different settings and then asked them which result was closest to their intention or which result was better.

Because the general methods used for travel planning are different from the method we proposed, we established a separate method for travel planning using the most common strategy. This method only prepares travel itineraries using highly popular tourist spots. To verify the objectivity of our proposed method, we recruited 50 college students between 23 and 25 years old to test it. They were asked to evaluate which travel itinerary planning method they preferred.

Each participant was asked to register on our system and to fill out the preference scores for each tourist spot property. The purpose of this step was to simulate a situation in which the participant had already been using the system for a while, so that the preference scores in the database would be approaching the actual preference scores of the participant. In addition, to reduce unnecessary variables, the departure point, the final destination, the number of travel days, and the start time of the tour were fixed for every participant. The departure point was Chang Gung University in Taoyuan for all cases, the destination was Feng Chia University in Taichung, and the number of travel days and the start time of the journey were two days and 9:00 am, respectively. We showed the results for travel planning based on only the popularity factor and on all factors and asked the users to select which result was better without informing them of the relationship between results and methods. As shown in Table 4, among the 50 participants, 35 preferred the method that used all factors; only 12 preferred the travel planning method based on popularity, and the remaining three were neutral regarding the two methods. According to Table 4, 70% of the participants preferred our proposed method for travel planning, indicating that our approach is attractive to most people and that it can effectively solve many of the current travel problems of Taiwanese vacationers.

In addition, 17 college students with an average age of 23 to 25 were recruited to verify whether the system is able to learn user preferences and to change the travel itinerary according to these preferences. This experiment was designed to determine whether the system can learn the preferences of a user after he or she has been using the system frequently and whether it can generate a travel itinerary based on these preferences. The participants were asked to register on the system at the beginning of the experiment. Unlike the last experiment, the participants were not required to input preference scores for each type of tourist spot. The itinerary was arranged using the initial preference scores assigned by the system. The participants were asked to set up six itineraries and to remove any tourist spots that did not interest them from the itineraries generated by the system. Among these six travel itineraries, the first and the sixth itineraries were used as the experimental group and the control group, respectively, and the departure points and destination points of these two tours were identical, that is, departure from Chang Gung University and arrival at Feng Chia University. The departure points and the destinations of the remaining four tours were selected by the participants. Similarly, to reduce unnecessary variables, the departure time and the number of travel days for these six tours were fixed at 9:00 am and one day, respectively. The travel itinerary of the experimental group was generated by the system before the system understood the preferences of the newly registered users, while the travel itinerary of the control group was generated by the system after repeated travel planning by the users with the system. Each of the 17 participants was asked to set up six travel itineraries using our system and to indicate whether the resulting itinerary for the control group or the experimental group was closer to their own preferences.

The results obtained in this experiment are shown in Table 5. The system was able to learn the preferences of each participant and to modify the travel arrangements according to user preferences. Among the 17 participants, 14 agreed that the tour plans arranged by the system after several itinerary planning sessions were closer to their favorite travel style compared to the first travel plan arranged after the initial registration. Only three participants favored the travel plans arranged by the system at the beginning.

When asked why they chose the initial travel plans arranged by the system, these three participants indicated they could adjust to a wide range of travel styles and that these plans happened to include many tourist spots of interest to them. For users who like all types of travel, the factors they consider in selecting an itinerary are different from those considered by users with narrower interests. Users who are less interested in particular travel modes will first be concerned about whether they are interested in all the properties of the tourist spots and will then consider whether the tourist spots that match their own preferences are popular. Conversely, those users who are satisfied with any travel mode will find any travel arrangement acceptable, regardless of the characteristics of the travel type; therefore, when selecting tourist spots, this type of user will be more inclined to select those that are more popular, which are the well-known tourist spots. The initial preference scores of these 17 newly registered users were given by the system. When the preference scores are the same, the itinerary arranged by the system is based on tourist spot popularity and user's concerns regarding cost. If the degree of concern regarding cost is the same, the itinerary arranged by the system will be solely based on the popularity of the tourist spots. Thus, the travel schedule created by the system for a newly registered user is based on the popularity of the tourist spot and user's degree of concern regarding cost.

Therefore, the three participants in the experiment who preferred the experimental group itinerary over that of the control group gave a higher priority to whether the tourist spots were popular than to whether the characteristics of the tourist spots were favorable, as considered by the other 14 participants. For these three participants, the average popularity of the tourist spots in the first tour itineraries arranged by the system was higher than that in the itinerary arranged by the system after learning users' preferences; therefore, the three participants preferred the first travel itinerary generated by the system. Because our system considers a variety of factors in making travel arrangements and presents the best result to the user, the average popularity of the tourist spots in the first tour itinerary arranged by the system is higher than that in the itinerary arranged by the system after learning user's preferences. After several planning sessions, the system will begin to consider a variety of factors during travel scheduling and will inevitably select between various factors. At this point, the average popularity of the tourist spots becomes only one of the considered factors, so the travel itinerary arranged by the system after learning user's preferences may assign a lower value to popularity than in the initial session.

## 5. Conclusions

Leisure travel has recently become a topic of great interest to Taiwanese residents. Most residents expect to be able to relax on a vacation trip during the holidays; however, the complicated arrangements involved with planning a travel itinerary are often discouraging, and, consequently, they often abandon the idea of traveling. In addition, the high price of a travel itinerary provided by a travel agency can also deter people from travelling. Therefore, we propose a new method that considers all factors affecting the travel preferences of users in planning a travel itinerary. The approach effectively combines the five most important factors considered by a traveler. It then uses a greedy algorithm to select the tourist spot with the highest score within a defined radius from the current position as the next destination. Thus, a travel itinerary matching the preferences of the user will automatically be generated. With this method, backpackers who want to go on a self-guided trip can easily plan their own travel route.

According to the results of the questionnaire assessment shown in Table 4, a small portion of participants preferred a tourist spot selection method based solely on popularity. After further inquiry regarding the reason for these results, most of the participants who preferred this method found those tourist spots rated the most popular to be the most interesting to them; thus, this method of tourist spot selection was preferable for them. However, if the method only considers popularity, travel scheduling requires a great deal of effort, and users may encounter negative situations, such as a destination that is closed, if the hours of operation are not taken into account. The participants who preferred the method used in our system all felt that the tourist spots selected by the system were closer to their personal preferences and that the resulting itinerary was planned very smoothly.

It is important for ATIPS to gather complete and relevant information on tourist spots. We will therefore continue working on automatically generating information, which was not the main goal of this portion of the research, in the next steps.

## Figures and Tables

**Algorithm 1:**
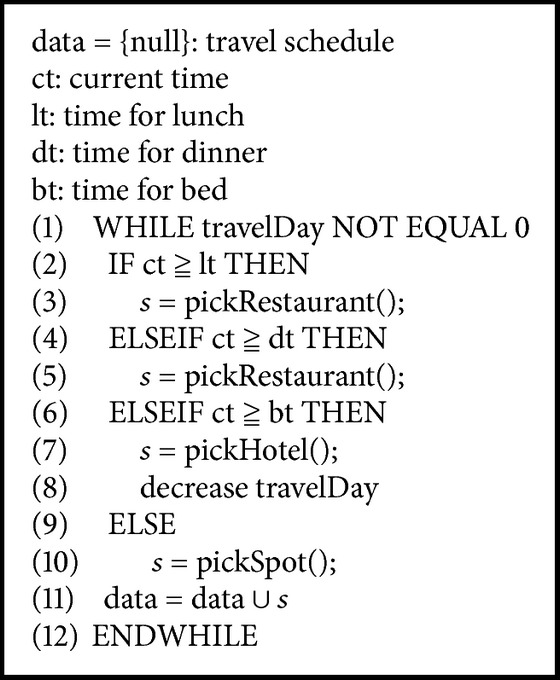
Travel scheduling algorithm.

**Algorithm 2:**
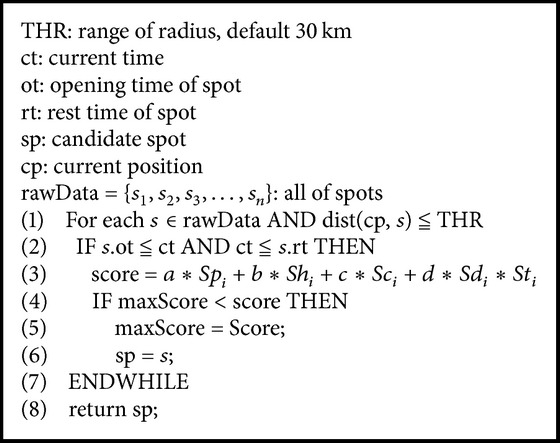
Tourist spot selection algorithm.

**Table 1:** Information store in our database of tourist spots.

Field Name	Description
SpotName	Name of the tourist spot
Occurrence	The frequency of occurrence from query results in search engines
Popularity	Score from the normalized occurrences from different queries
Characteristic	Characteristics of the tourist spot
isRestaurant	Category of the tourist spot (whether it includes a restaurant)
isAccommodation	Category of the tourist spot (whether includes accommodations)
Cost	Cost score
TimeOpen, TimeClose	Hours of operation
Latitude, Longitude	Latitude and longitude of the tourist destination in the GPS

**Table 2:** Comparison of various software options for travel planning.

Name of software	Summary	Advantages/disadvantages
Niceday [17]	A web service that recommends tourist spots and arranges itineraries according to users' selected spots. The website provider recommends tourist spots to users.	*Advantages*: (i) Automatic path planning (ii) Sharing itinerary planning (iii) Editors' recommended spots *Disadvantages*: (i) Manually selecting spots (ii) Manually selecting the time period for each spot (iii) No preferences

Funlidays [18]	A mobile application that provides an introduction to tourist spots worldwide. It also provides itinerary planning according to users' selected spots. Funlidays will recommend nearby spots to the user for planning.	*Advantages*: (i) Recommending nearby spots (ii) Automatic path planning (iii) Offline information on spots *Disadvantages*: (i) Manually selecting spots (ii) Manually selecting the time period for each spot (iii) No preferences

KuruMap [19]	A web service that provides information regarding tourist spots and activities. It also provides an itinerary planning tool and a blog. Users need to include the tourist spots in “my favorites” for travel planning.	*Advantages*: (i) Recommending spots using social networks (ii) Nearby traffic information (iii) Forum to discuss spots *Disadvantages*: (i) Manually selecting spots (ii) Manually selecting the time period for each spot (iii) No preferences

NAVIG [20]	An artificial system that provides navigation guidance for those visually impaired people. With real-time image recognition and GPS position calculation, the system assists spatial audio visually impaired people reaching their destination.	*Advantages*: (i) Automatic path planning (ii) Spatial audio navigation guidance (iii) Indoor navigation with building map *Disadvantages*: (i) No spot information (ii) No time period for each spot to select (iii) No preference

BlindNavi [21]	An artificial app. that provides navigation guidance for those visually impaired people. With two input methods, voice and text, and two notification methods, voice and vibration, this system assists visually impaired people	*Advantages*: (i) Automatic path planning (ii) Audio navigation guidance and vibration interaction (iii) Sharing walking experience and (iv) Microlocation information *Disadvantages*: (i) No time period for each spot to select (ii) Extra sensors deployed (iii) No preference

HEARE [22]	An artificial app. that provides navigation guidance for those visually impaired people. People create routes from website and share them. By using provided app., others can use those created routes to walk.	*Advantages*: (i) Automatic path planning (ii) Spatial audio navigation guidance (iii) Sharing itinerary planning *Disadvantages*: (i) Manually selecting spots (ii) No spot information (iii) No time period for each spot to select

Our proposed, ATIPS	A web service that provides automatic itinerary planning according to multiple factors based on just the departure and destination points. It also automatically learns the user preferences during the interactive itinerary adjustment.	*Advantages*: (i) Automatic itinerary planning (ii) Automatic preference learning (iii) Automatically recommending spots *Disadvantages*: No traffic information

**Table 3:** Average score comparison between popularity factor and all factors.

	Based on popularity (average score)	Based on all factors (average score)
From Chang Gung University to Feng Chia University	3.72	128.11
From Ocean University to the Hsinchu County Hall	4.54	76.82
From National Taipei University of Technology to Taichung Railway Station	3.25	128.94
From Hsinchu Railway Station to Tunghai University	45.68	82.04
From Keelung High School to Taichung Railway Station	2.78	180.67
Average	11.99	119.32

**Table 4:** Evaluation results comparing satisfaction between user preferences and popularity.

	Number of people	Percentage (%)
Based on user preferences	35	70
Based on popularity	12	24
No opinion	3	6
Total number of people	50	100

**Table 5:** Evaluation results comparing satisfaction between before and after travel itinerary planning using the ATIPS learning mechanism.

	Number of people	Percentage (%)
Before preferences learning	3	7.65%
After preferences learning	14	82.35%
Total number of people and percentage	17	100%
